# Protocol for Construction of Rat Nerve Stimulation Cuff Electrodes

**DOI:** 10.3390/mps2010019

**Published:** 2019-02-15

**Authors:** Manolo U. Rios, Jesse E. Bucksot, Kimiya C. Rahebi, Crystal T. Engineer, Michael P. Kilgard, Seth A. Hays

**Affiliations:** 1 School of Brain and Behavioral Sciences, The University of Texas at Dallas, Richardson, TX 75080-3021, USA; manolo.rios@utsouthwestern.edu (M.U.R.); kilgard@utdallas.edu (M.P.K.); seth.hays@utdallas.edu (S.A.H.); 2 School of Biomedical Engineering, The University of Texas at Dallas, Richardson, TX 75080-3021, USA; jesse.bucksot@utdallas.edu; 3 Texas Biomedical Device Center (TxBDC), The University of Texas at Dallas, Richardson, TX 75080-3021, USA; kimiyarahebi@gmail.com

**Keywords:** vagus nerve stimulation, vagus nerve, cuff electrode, micro-construction, vagal nerve

## Abstract

Peripheral nerve stimulation has emerged as a platform therapy to treat a wide range of disorders. Continued development and translation of these strategies requires that researchers have access to reliable, customizable electrodes for nerve stimulation. Here, we detail procedures to build three different configurations of cuff electrodes with varying numbers and orientations of contacts for nerve stimulation in rats. These designs are built with simple, widely available materials, using platinum–iridium electrodes assembled into polyurethane tubing. Moreover, the designs can easily be customized to increase versatility and individualize for specific stimulation applications. This protocol provides a resource to facilitate the construction and customization of stimulation cuffs to support preclinical nerve stimulation research.

## 1. Introduction

Peripheral nerve stimulation has emerged as a strategy to treat a wide range of neurological disorders. Vagus nerve stimulation (VNS) is one of the most widely used applications and has been used in over 70,000 patients for intractable epilepsy [1]. Recent studies indicate that VNS may be useful for other disorders, including stroke, tinnitus, and PTSD [2,3,4,5,6,7,8,9,10,11,12,13,14,15]. In addition to VNS, other peripheral nerve stimulation therapies include the use of tibial nerve stimulation for bladder control and occipital nerve stimulation for migraines [16,17]. Preclinical development is critical to the effective clinical translation of these emerging therapies. However, broad access to reliable electrodes for nerve stimulation in animal models can limit the ability of researchers to carry out these preclinical studies.

Here, we present a detailed protocol for the micro-construction of different implementations of cuff electrodes for peripheral nerve stimulation to increase access for researchers. The cuffs are similar in design to the standard configurations used in most preclinical studies and are constructed using widely available materials and simple techniques. The use of a microscope aids in increasing standardization across cuffs, quality, and durability. While the cuff electrode design will ultimately depend on the details of each experimental preparation, we describe the construction of a widely-applicable, simple, two-channel circumferential cuff electrode. This cuff design has been employed in a number of studies for vagus nerve stimulation in rats and shares the majority of features with commercially available cuffs from MicroProbes and CorTec [2,3,4,5,6,7,10,12,13,14,15,18,19,20,21,22,23,24]. Additionally, we provide designs for two additional multichannel cuff electrode configurations which can be modified to suit a greater range of preparations. Finally, we describe two methods to test the efficacy and reliability of nerve stimulation. This protocol provides a framework for researchers to construct reliable peripheral nerve stimulation cuff electrodes for a variety of applications to support the preclinical development and translation of nerve stimulation therapies. 

## 2. Experimental Design

Below, we provide a detailed description of the methodology to construct three cuff designs for nerve stimulation in rats. The designs described here vary in length, number of stimulating electrodes, construction times, and required tools and materials. Table 1 provides a complete list of the items necessary to create all cuffs while the components of all cuffs are presented in Table 2. The detailed steps to construct each cuff design are documented in the Procedure Section below. The University of Texas at Dallas Institutional Animal Care and Use Committee approved all surgical protocols and recording procedures (protocol #14-10, date of approval 08/13/2018).

### 2.1. Evaluation of Stimulation Efficacy

#### 2.1.1. Vagus Nerve Stimulation

All handling, housing, stimulation, and surgical procedures were approved by The University of Texas at Dallas Institutional Animal Care and Use Committee. Rats were anesthetized with isoflurane (1–3%) in 0.5 L/min air and placed on a heating pad (Stryker, T/Pump). An incision and blunt dissection of the muscles in the neck exposed the left cervical vagus nerve, according to standard procedures [6,23,25]. The nerve was placed into the cuff electrode, and the leads were connected to an isolated pulse stimulator (A-M Systems, Model 4100). Cuff impedance was monitored using an oscilloscope (Pico Technology, PicoScope 2204A). Rapid stimulation-dependent reductions in blood oxygen saturation, which report vagal activation via the Hering–Breuer reflex [26], were recorded using a pulse-oximeter (Starr Life Sciences, MouseOx Plus). Blood oxygen saturation was sampled at 10 Hz. Data was filtered using a 10-sample moving average filter. Stimulation consisted of 10-second trains of biphasic pulses (100 µs pulse width) at 30 Hz and 0.8 mA. Stimulation was delivered every 60 s, but was delayed if needed to allow oxygen saturation to return to baseline. 

#### 2.1.2. Sciatic Nerve Stimulation

Rats were anesthetized using ketamine hydrochloride (80 mg/kg, IP injection) and xylazine (10mg/kg, IP). Once the surgical site was shaved, an incision was made on the skin directly above the biceps femoris muscle. The sciatic nerve was exposed by dissecting under the biceps femoris. The gastrocnemius muscle was separated from skin and surrounding tissue using blunt dissection. Cuff electrodes were then placed on the sciatic nerve with leads connected to an isolated programmable stimulator (Model 4100; A-M Systems™; Sequim, WA, USA). The nerve was left in place underneath the gastrocnemius. The cavity was kept full of saline at all times to ensure that the cuff would be operating in a uniform medium with conductance similar to tissue. The Achilles tendon was severed at the ankle and affixed to a force transducer using nylon sutures. The foot was clamped and secured to a stereotaxic frame to prevent movement of the leg during stimulation and to isolate recordings from the gastrocnemius muscle. 

Stimulation consisted of 0.5 s trains of biphasic pulses (100 µs pulse width) at 30 Hz with varying current amplitudes delivered using the isolated pulse stimulator. Stimulation intensities were randomly interleaved. Voltage was recorded using a digital oscilloscope (Pico Technology, PicoScope 2204A). The force of muscle contraction was sampled at 10 Hz and recorded through a force transducer which was connected to an analog channel on an Arduino Mega 2560. All components were integrated using MATLAB. Stimulation was delivered every 15 s and each parameter was repeated in triplicate. 

## 3. Procedure

### 3.1. Preparation of Tools

Drilling Tool (5 min). This section details the process of making a tool to drill holes in the polyurethane tube, through which leads and silk thread will be passed to construct the cuff. Handle the sharp edge with caution. Repeat the third and fourth step in this section to sharpen the drilling tool as needed during the micro-construction procedure (approximately every two uses).

**Figure 1:**
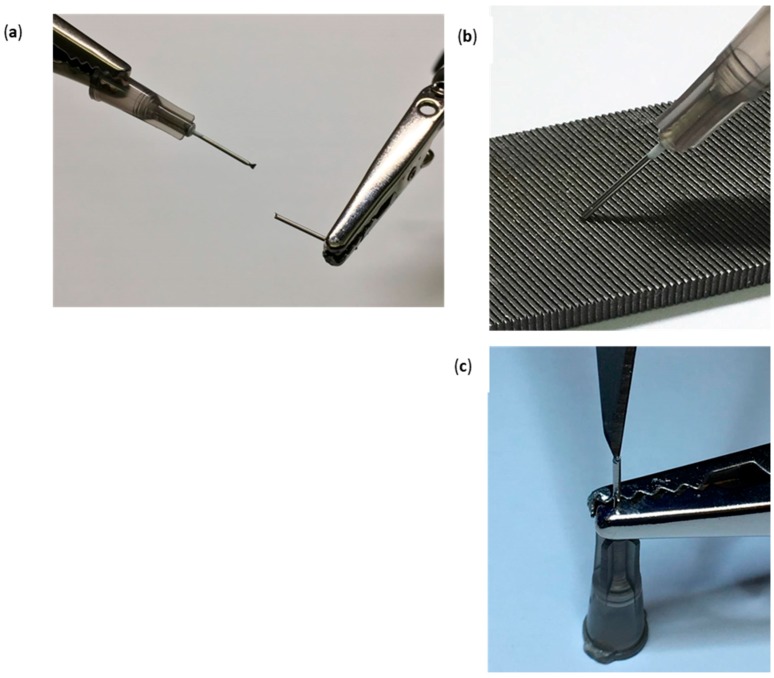
Drilling Tool; (**a**) 1.5 cm shaft removal from the 22G 1” needle using wire cutters and helping hands. (**b**) Sharpening of the drilling tool by rotating its flat edge across a nail file in a 45° angle. (**c**) Residue removal from the drilling tool bevel using a #11 scalpel blade.

1 Hold the ends of a 22G 1” needle using the alligator clips of two helping hands and cut 1.5 cm of the needle shaft using wire cutters (Figure 1a). Dispose of the sharp needle tip in a sharps container.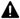 **CRITICAL STEP** Use caution when handling either end to prevent injuries.
2 Hold the tip of the needle perpendicular to the surface of a metallic file and file the tip of the needle until completely flat.
3 Hold the flat needle tip against the metallic nail file at a 45° angle and move the needle sideways while rotating it to sharpen the edges (Figure 1b).
4 Place the needle under a microscope (lumen facing up) and remove any metallic residues at the lumen by inserting and rotating the tip of the #11 scalpel blade into the flat bevel (Figure 1c).

#### 3.1.1. Torch Setup (5 min)

This section details the process of setting up a jewelry torch, which is necessary to construct the wires which are for the stimulation contacts in the cuff.


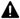
**CRITICAL STEP** Use caution and follow manufacturer instructions when setting up the torch.

1 Connect the oxygen and propane preset regulators to the fuel hoses of the jewelry torch by capping their adapters.
2 Insert the #3 tip at the top of the torch handle.
3 Connect the propane and oxygen tanks to their respective preset regulators.
4 Turn the valves located on the preset regulators to open the oxygen and propane flow.

#### 3.1.2. Surface Aid (Optional)

For better handling of the small components we recommend the creation of a small surface aid. Objects such as needles, tape, and the tubing can be easily attached to this surface.

1 Obtain a flat piece of cardboard and fold it over itself to create a small cardboard square or a rectangle.
2 Wrap the entire folded cardboard with masking tape to prevent unfolding. Wrapping the folded cardboard with a total of two or three layers of tape is recommended.

### 3.2. Standard Circumferential Cuff Electrode

**Figure 2:**
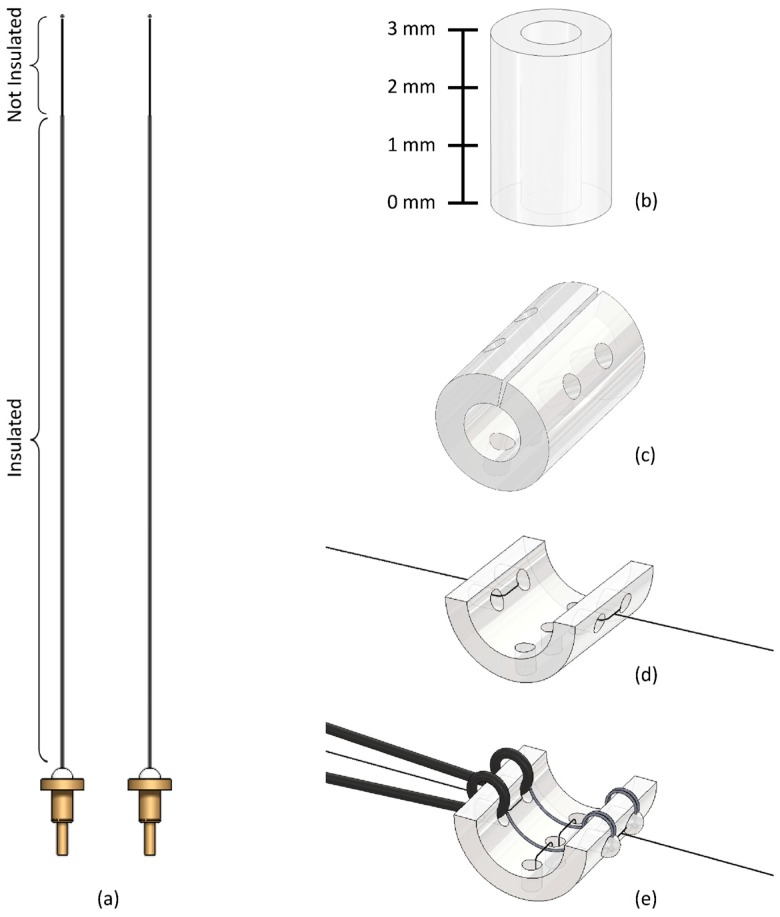
Standard Cuff Micro-Construction Flowchart. (**a**) Detail of the platinum–iridium multistranded wires before insertion showing the portion of the wire where insulation has been removed. The press-fit gold pin connectors to allow connection of the cuff to a headmount are shown. (**b**) 3 mm segment of Micro-Renathane (Polyurethane) Tubing. (**c**) Nerve cuff skeleton with anterior cut and all holes drilled. (**d**) Nerve cuff skeleton with silk knots attached. The cuff is shown pulled open by the silk threads. (**e**) Standard nerve cuff finished product. The inner PI wires are affixed to the bottom of the cuff with silk thread, and acrylic is applied to cover the holes in the cuff.

A detailed description of the Standard Circumferential Cuff Electrode (Figure 2) construction process is presented below.


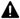
**CRITICAL STEP** All procedures in this section must be performed under a microscope in order to maximize the advantages of this protocol.

**Figure 3:**
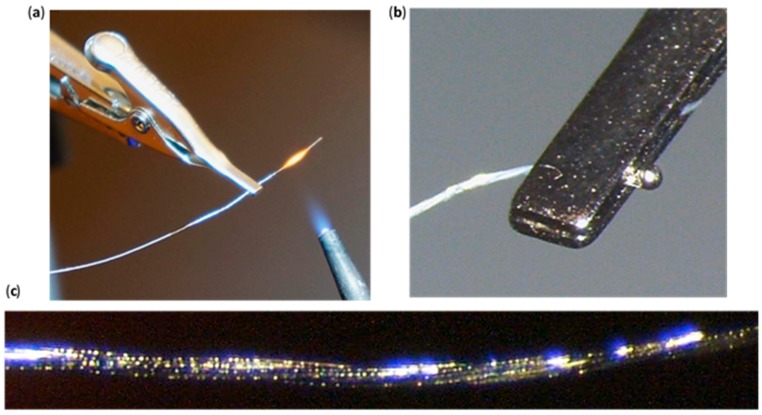
PI Wire; (**a**) Sharp blue flame of the jewelry torch (propane and oxygen) passed across the clamped 1cm PI wire (approximately 1 cm away). PI wire insulation is burned off and individual wires are fused together. (**b**) PI wire “big ball end”. Melted 8 mm of PI wire into a ball using the sharp blue flame of the jewelry torch. (**c**) Wire melting indicated by bubbles throughout the entire 1 cm PI wire.

**Figure 4:**
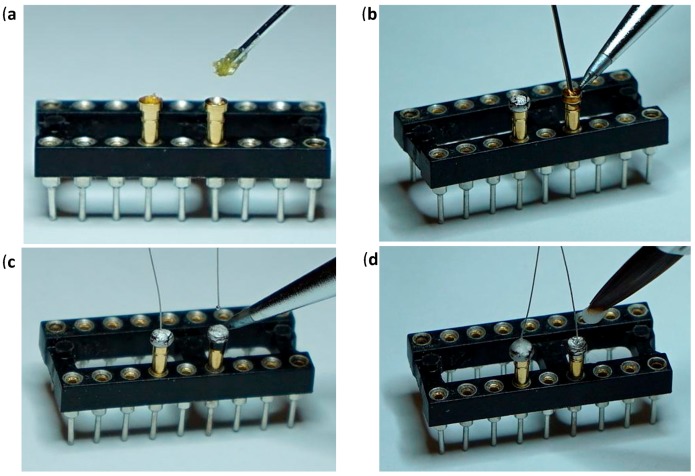
Gold Pin Wire; (**a**) Insertion of the appropriate number of gold pins to the dip socket and application of liquid paste flux to the gold pin mounting holes. (**b**) Gold pin mounting holes filling with lead-free solder. (**c**) Solder re-melting and insertion of the 6.9 cm PI wires. (**d**) Acrylic covering of gold pin top end.

**Figure 5:**
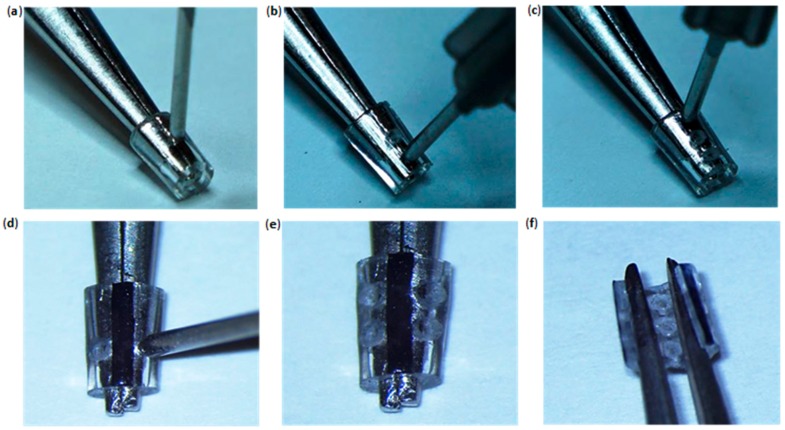
(**a**) Middle posterior hole displayed medially at a 1.5 mm length. (**b**) Lower posterior hole displayed medially at a 0.5 mm length (1 mm below the middle posterior hole). (**c**) Upper posterior hole displayed medially at a 2.5 mm length (1 mm above the middle posterior hole). (**d**) Right and left inferior holes displayed 45° away from the 0° line respectively. (**e**) Addition of the right and left superior holes displayed 45° away from the 0° line respectively. (**f**) Anterior cut across the 0° line. Posterior holes are now visible through the opening.

**Figure 6:**
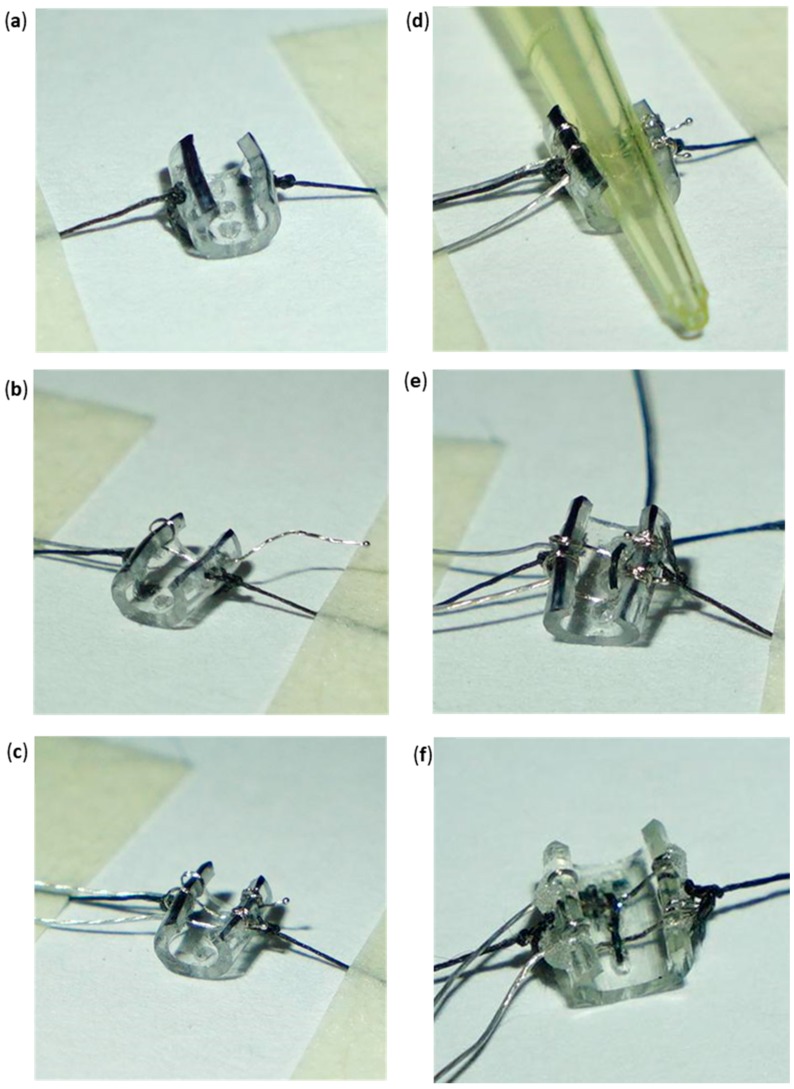
(**a**) Cuff skeleton secured through lateral silk strands to expose the inside of the tube. (**b**) PI wire with gold pins looped once at the left superior hole and moved across the right superior hole. Only uninsulated PI wire exists inside of the tube. (**c**) Gold pin wires assembled into their respective locations. PI wires have been looped twice at the right superior and inferior holes. (**d**) Pressing of the two looped gold pin wires using a 10 µL pipette tip against the inside wall of the cuff skeleton. (**e**) Silk “D Loop” formed between the upper and middle posterior holes. (**f**) Completed standard cuff. Inside silk knotting follows a zigzag pattern. Minimal application of acrylic is desired.

1 **Preparation of Platinum-Iridium Wires (10–15 min)**. Place a helping hand at the edge of a flat working surface and secure it with a clamp. Then, switch the regular alligator clip for a micro-alligator clip.
  1.1 Looking under the microscope, use a ruler to measure 1 cm of platinum–iridium (PI) wire and hold it in the micro-alligator clip. Do not detach or cut the wire from the wire spool.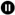 **PAUSE STEP** Flammable materials required ahead. Previous experience using a jewelry torch is recommended. Use caution working with the torch. Do not ignite the pilot flame pointing at yourself or other individuals.
  1.2 Slightly open the propane valve at the torch handle and ignite the pilot flame.
  1.3 Slightly open the oxygen valve at the top of the jewelry torch until the pilot flame has turned into a sharp blue flame. Opening the valve too fast or too much might cause a loud popping sound. If this occurs, close the oxygen valve, reignite the pilot flame, and repeat this step.
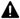
**CRITICAL STEP** Keep the blue flame approximately 1 cm away from the wire when performing Step 1.4. Passing the blue flame too close to the PI wire will result in a wire kink that will damage the wire and irritate the vagus nerve after implantation.
  1.4 **PAUSE STEP** Performing Steps 1.4 and 1.5 will heat the micro-alligator clip and the PI wire. Always allow approximately 1 min for cool down before touching the clip and the wire after these steps.
    Looking under the microscope, point the tip of the blue sharp flame to the 1cm PI wire end and pass it all the way across without touching it (approximately 1 cm away from the wire), heating it to a bright orange. Repeat this several times until the wires have fused together which will be indicated by the formation of bubbles in the PI wire (Figure 3a,c).
  1.5 Looking under the microscope, touch the end tip of the clamped 1 cm PI wire with the sharp blue flame to melt 1 mm of the PI wire into a ball. We will refer to the small ball and the fused 9 mm of wire collectively as the “small ball end”.
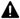
**CRITICAL STEP** Close the oxygen and propane valves at the handle of the jewelry torch. Place the jewelry torch in a secured location until further indication. Release the PI wire from the alligator clip and use a ruler to measure 7.7 cm of PI wire from the melted ball of the small ball end to the spool of PI wire. Then, cut the wire at that location using the surgical scissors.
  1.6 Use a ruler to measure 8 mm of PI wire at the insulated end of the 7.7 cm PI wire (opposite from the small ball end). Secure the wire in the micro-alligator clip.
  1.7 Completely melt the 8 mm of PI wire into a ball by re-igniting the jewelry torch and touching the 8 mm PI wire end with the sharp blue flame. We will refer to this melted section as the “big ball end”. Completion of Steps 1–1.8 in this section will create a 6.9 cm wire with a big ball end, a 6 cm insulated section (middle portion), and a small ball end (9 mm of fused wire with small ball) (Figure 3b).
  1.8 Repeat steps 1–1.8 in this section to create a second standard cuff PI wire.
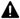
**CRITICAL STEP** Steps 1.10–1.16 must be performed under the microscope.
  1.9 Insert two gold pins into the female end of the dip socket.
  1.10 Use a paper clip to add a miniscule dab of water-soluble flux paste into the mounting holes of the four gold pins (Figure 4a).
  1.11 Set the temperature of the soldering iron to 700 °F and allow it to heat (30–60 s) then, touch the top edge of each gold pin for approximately two seconds to melt the flux paste into the mounting holes. Repeat this step a couple more times to make sure the water-soluble flux has covered the entire inside of the gold pins.
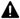
**CRITICAL STEP** Clean the soldering iron tip as needed using a wet sponge.
  1.12 One at a time, insert the solder wire into the gold pin mounting hole and touch the upper edge of the gold pin to melt the solder. Completely fill the mounting hole with the lead-free solder (Figure 4b).
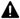
**CRITICAL STEP** No solder must drip from the mounting hole. If this occurs, replace that gold pin for a new one and repeat Steps 1.11–1.13.
  1.13 Use the tip of the #5 jeweler’s forceps to clean any excess solidified flux paste at the top of the gold pins.
  1.14 One at a time, touch the top edge of each gold pin with the soldering iron to re-melt the solder inside the mounting holes. At the same time, insert the 3 mm uninsulated end of the 6.9 cm PI wires (one for each gold pin). Then, remove the soldering iron from the gold pins and allow the solder to solidify with the 3 mm uninsulated ends inside the mounting holes (Figure 4c).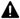 CRITICAL STEP Keep the wire straight and steady when inserting the big ball end of the PI wires into the mounting holes to prevent any damage to the insulation not being inserted.
  1.15 Apply a tiny drop of acrylic (liquid acrylic mixed with powder acrylic) at the top of each gold pin using a fine detail brush (Figure 4d).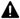 CRITICAL STEP No acrylic must drip off the top of the gold pins. If this becomes the case, use a small paper towel to wipe off the acrylic around the gold pin.
2 **Cuff skeleton (15–20 min)**. Use a ruler to measure 3 mm of polyurethane tubing and cut it using a single-straight scalpel cut.
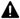
**CRITICAL STEP** The tube should have no rough edges at either end in order to prevent irritation after implantation.
  2.1 Insert the closed tips of a #5 jeweler’s forceps in the 3 mm polyurethane tube, lay them horizontally over your working surface, and place them perpendicular in respect to your shoulders. The side of the 3 mm tube facing upwards will be referred to as the posterior side.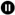 **PAUSE STEP** Dull forceps are recommended for safety.
  2.2 Press and rotate the flat bevel of your drilling tool against the surface of the 3 mm polyurethane tube at a 1.5 mm length (middle of the tube) to carve a hole (Figure 5a). This hole will be referred to as the middle posterior hole.
  2.3 Insert a 27G 1¼” needle through the hub of the drilling tool to remove any tubing stuck on the inside of the shaft.
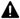
**CRITICAL STEP** Step 2.3 must be performed every time after a hole is drilled. Failure to do this will accumulate polyurethane residues inside the drilling tool, making carving at the surface of the tube extremely difficult.
  2.4 Use your drilling tool to drill a hole on the 3 mm polyurethane tube at a 0.5 mm length (1mm below the middle posterior hole). This hole will be referred as the lower posterior hole (Figure 5b). Sharpen the drilling tool if needed (see “Drilling Tool” in the Equipment Setup section).
  2.5 USe your drilling tool to drill a hole at a 2.5 mm length (1 mm above the middle posterior hole). This hole will be referred to as the upper posterior hole (Figure 5c). Completion of this step should create a 3 mm tube with three posterior holes (upper, middle, lower).
  2.6 Rotate the #5 forceps 180° in order to move the posterior side of the tube (with the posterior holes) face-down and the anterior side face-up.
  2.7 Use a fine-tip permanent marker to draw a straight line on the surface of the 3 mm tube directly across the three posterior holes (Figure 5d). This mark will be used to indicate a 0° line on the surface of the tube.
  2.8 Use your drilling tool to drill a hole at a 1mm length and 45° leftwards from the 0° mark. This hole will be referred to as the left inferior hole and the face-up surface of the 3mm tube will be referred as the anterior side. Sharpen the drilling tool if needed.
  2.9 Drill a hole at a 1 mm length and 45° rightwards from the 0° mark (mirror from the left inferior hole across the 0° line) using your drilling tool. This hole will be referred to as the right inferior hole.
  2.10 Drill a hole at a 2 mm length and 45° leftwards from the 0° mark. (1 mm above the left inferior hole) using your drilling tool. This hole will be referred to as the left superior hole. Sharpen the drilling tool if needed.
  2.11 Drill a hole at a 2 mm length and 45° rightwards from the 0° mark. (1 mm above the right inferior hole) using your drilling tool. This hole will be referred to as the right superior hole. The right and left inferior and superior holes will be collectively referred to as the lateral holes (Figure 5e).
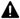
**CRITICAL STEP** Hold the #5 jeweler’s forceps inside the tube in a closed position with your hands, so they do not suddenly open when performing Step 2.12.
  2.12 Without changing the position of the 3 mm polyurethane tube, use a scalpel to make a straight cut along the entire 0° mark on the surface of the tube. This cut will be referred to as the anterior cut (Figure 5f). This is now a completed skeleton.
3 **Cuff assembly (15–20 min**). Thread 30 cm of silk into the eye of the #5 sewing needle.
  3.1 Pass the needle through the right inferior hole towards the inside of the skeleton. Leave approximately 3 cm of silk on the outside of the tube.
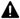
**CRITICAL STEP** The needle should not go through the left inferior or superior hole at any moment.
  3.2 Turn the needle 180° and pass it through the right superior hole from the inside to the outside of the skeleton thus forming a small “D” loop between the right superior and the right inferior hole at the inside of the skeleton.
  3.3 Make two ligature knots to tie the 3 cm silk end and the long silk end (Supplemental Video).
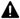
**CRITICAL STEP** All ligature knots must be firmly tied to prevent detachment. The ligature knots must rest between the superior and inferior holes.
  3.4 Measure 5 cm of silk from the skeleton to the end of the needle and make a cut at that location using the surgical scissors.
  3.5 Trim the 3 cm silk end to approximately 5 mm.
  3.6 Repeat Steps 3.1–3.5 with the left inferior and superior holes.
  3.7 Carefully pull each of the 5 cm silk strands in opposite directions to slightly open the inside of the skeleton. Then, use tape strips to attach each of the 5cm silk strands against a flat surface under the microscope (Figure 6a).
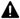
**CRITICAL STEP** The skeleton should lie with the anterior cut facing up (towards the microscope) and the posterior holes facing down (towards the flat surface).
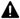
**CRITICAL STEP** The inside surface of the skeleton should be visible through the anterior cut.
  3.8 Pass the entire small ball end of one of the gold pin wires through the left superior hole from the outside of the tube to the inside of the tube until approximately 1 mm of insulated PI wire lies inside the tube.
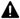
**CRITICAL STEP** No portion of the PI wire should go through the right superior or inferior holes during this step.
    Use the #5 jeweler’s forceps to grab the small ball at the gold pin wire small ball end and loop it around the left superior hole once (from the inside to the outside and to the inside again). Insulated wire should wrap the superior hole, but only uninsulated wire should re-enter the inside of the tube.
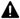
**CRITICAL STEP** The edge between the uninsulated and the insulated sections of the gold pin wire should rest at the left superior hole.
  3.9 Tighten the loop around the left superior hole by pulling both ends of the gold pin wire in opposite directions.
  3.10 Use the #5 jeweler’s forceps to pass the remaining portion of the small ball end through the right superior hole (from the inside to the outside of the skeleton) (Figure 6b).
  3.11 Insert a pipette tip through the anterior cut (parallel to the tube) to push the uninsulated wire inside the skeleton against the inner wall of the tube.
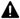
**CRITICAL STEP** The fused wire section of the small ball end touching the inner wall of the tube should run between the upper posterior hole and the middle posterior hole.
  3.12 Grab the small ball of the gold pin wire at the outside of the tube using two #5 jeweler’s forceps and loop it around the right superior hole twice.
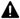
**CRITICAL STEP** Be extremely careful handling the small ball end when looping it twice. Rough handling may break or damage the wire thus compromising the cuff.
  3.13 Repeat Steps 3.4–3.13 with the second gold pin wire at the left and right inferior holes (Figure 6c,d).
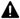
**CRITICAL STEP** The uninsulated section of the second gold pin wire inside of the skeleton should run between the middle posterior hole and the lower posterior hole.
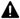
**CRITICAL STEP** Once the gold pin wiring has been completed, make sure the uninsulated sections of the wire inside the skeleton are not damaged (broken, fried, dented, etc.). If damaged, remove the entire gold pin wire and replace it with a new one.
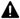
**CRITICAL STEP** Both sections of the uninsulated gold pin wires inside the skeleton must run against the inner wall.
  3.14 Turn the skeleton to a vertical position without removing the tape holding the 5 cm silk strands.
  3.15 Pass the #5 sewing needle with silk through the upper posterior hole from the outside of the skeleton towards the interior of the skeleton leaving 3 cm of silk behind.
  3.16 Turn the needle 180° and pass it through the middle posterior hole. Pull the silk until a “D” loop is formed on the inside of the wall between the upper and middle posterior holes (Figure 6e).
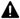
**CRITICAL STEP** The “D” loop formed between the upper posterior hole and the middle posterior hole should pull the uninsulated gold pin wire between these holes against the inner wall of the tube.
  3.17 Turn the needle 180° and pass it through the lower posterior hole towards the inside of the skeleton.
  3.18 Turn the needle 180° and pass it through the middle posterior hole. Pull both ends of the silk to form a “D” loop between the lower and middle posterior holes at the inside of the tube.
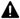
**CRITICAL STEP** Similarly to Step 3.16, the “D” loop formed between the lower posterior hole and the middle posterior hole should pull the uninsulated melted section of the gold pin wire between these holes against the inner wall of the tube.
  3.19 Make three ligature knots using the 3 cm silk end and the silk with the needle end on the outside of the skeleton (posterior side). Then, remove the tape from the 5 cm silk strands to release the skeleton.
  3.20 Use the fine-tip acrylic brush to add a miniscule drop of wet acrylic to each of the lateral holes and the lateral ligature knots. Wait 5 min for the acrylic to dry before touching the now completed VNS cuff.
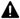
**CRITICAL STEP** Make sure no acrylic drips inside of the skeleton. Clean the inside of the skeleton immediately with a surgical spear (Weck-Cell) wet in 70% isopropyl rubbing alcohol if this becomes the case.
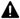
**CRITICAL STEP** Failure to maintain an acrylic-free environment inside the skeleton could affect the reliability of stimulation by electrode-nerve contact obstruction.

This is now a completed Standard VNS cuff (Figure 6f).

### 3.3. Longitudinal Circumferential Cuff

**Figure 7:**
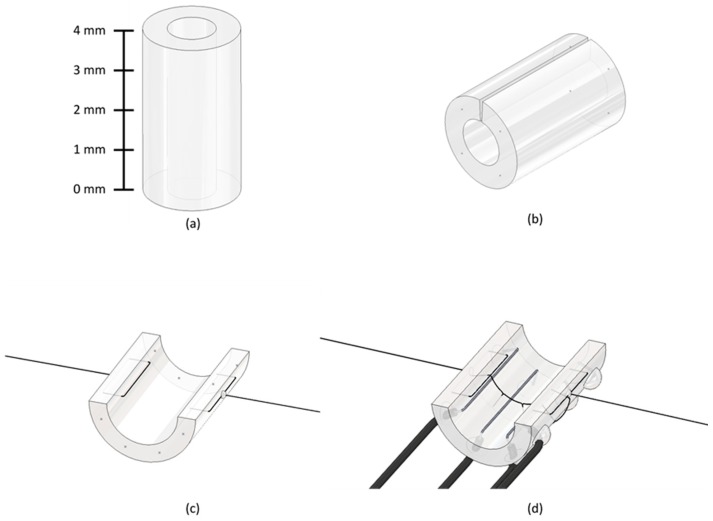
Longitudinal Cuff Micro-Construction Flowchart. (**a**) 4 mm segment of polyurethane tubing. (**b**) Anterior cut and four marks at each of the tube’s edges. (**c**) Cuff skeleton depicting insertion of silk threads and application of UV adhesive at the thread insertion point. (**d**) Completed longitudinal cuff, with insertion of four PI wires. The uninsulated portion of PI wire is located on the interior of the cuff to allow stimulation and is secured with silk thread, while the insulated portion of the PI wire is outside of the cuff and insertion points are secured with UV adhesive.

A detailed description of the Longitudinal Circumferential Cuff Electrode (Figure 7) construction process is presented below.

**Figure 8:**
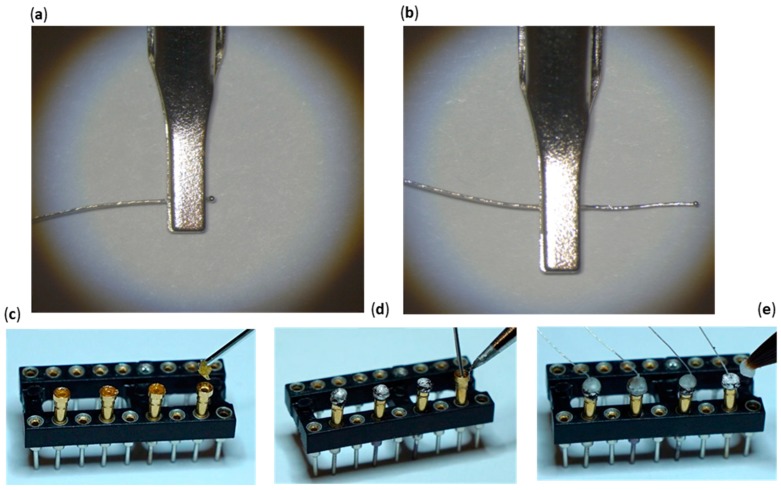
Longitudinal Gold Pin Wires; (**a**) Big ball end of the platinum–iridium (PI) wire for the longitudinal cuff. (**b**) Small ball end of the platinum–iridium wire for the longitudinal cuff. (**c**–**e**) Longitudinal cuff gold pin wire assembly using water-soluble flux paste (**c**), lead-free solder (**d**), and acrylic (**e**).

**Figure 9:**
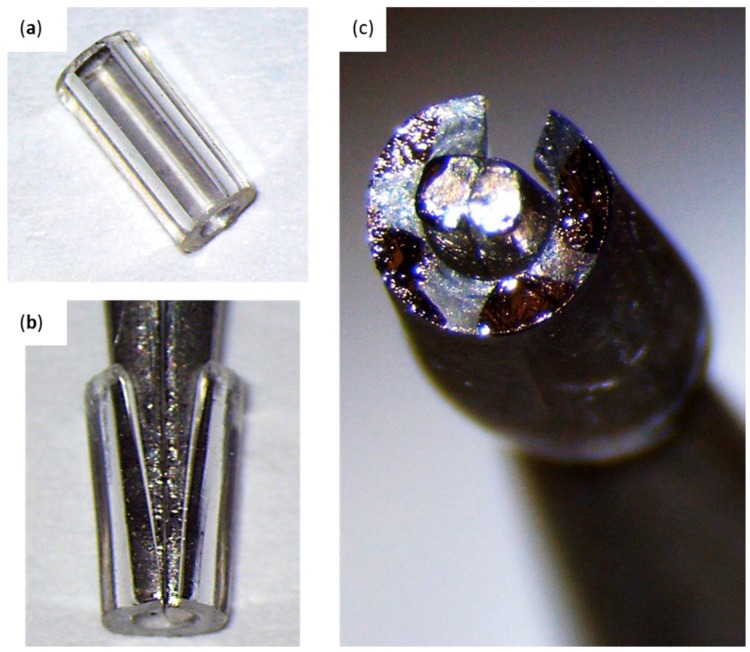
Longitudinal Cuff Skeleton Construction; (**a**) Micro-polyurethane tubing for longitudinal cuffs (4 mm length). (**b**) Anterior cut creation using the space between forceps along the entire tube’s length. (**c**) Marks A (first quadrant), C (fourth quadrant), E (third quadrant), and G (second quadrant) created.

**Figure 10:**
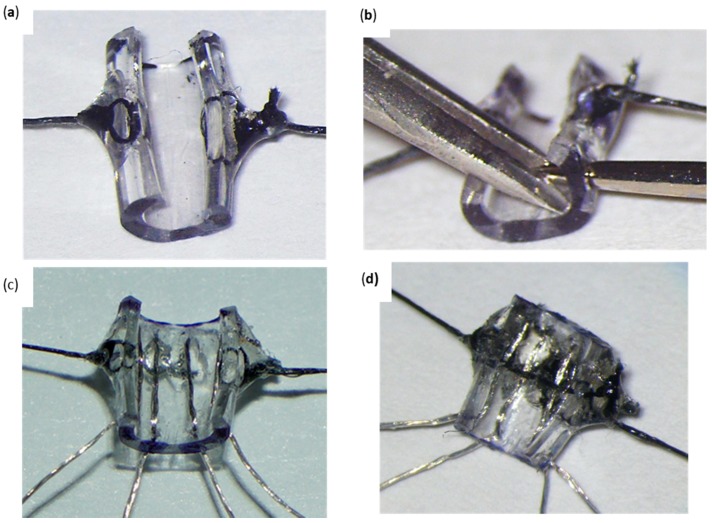
Longitudinal Cuff Assembly; (**a**) Lateral silk knotting to expose the inside of the micro-polyurethane tube. (**b**) Piercing of the exterior surface of the tube (0.5 mm above Mark A) using a needle. The needle will eventually make a U-turn and pierce the interior surface of the tube 0.5 mm below mark B. (**c**) Longitudinal PI wire leads running along the length of the micro-polyurethane tube. (**d**) Completed longitudinal cuff. Longitudinal leads along the length of the micro-polyurethane tube are pressed against the inside wall of the tube by silk strands and secured with UV glue.

1 **Gold Pin Wires (10 min)**. Looking under the microscope, use a ruler to measure 3 mm of platinum-iridium (PI) wire and hold it in the micro-alligator clip. Do not detach or cut the wire from the wire spool.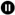 **PAUSE STEP** Flammable materials required ahead. Previous experience using a jewelry torch is recommended. Do not ignite the pilot flame pointing at yourself or other individuals.
  1.2 Slightly open the propane valve at the torch handle and ignite the pilot flame by placing the flame of a lighter near the tip of the torch.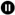 **PAUSE STEP** Performing Step 1.3 may dangerously heat the micro-alligator clip and the PI wire. Always allow approximately 1 minute for cool down before touching the clip and the wire after these steps.
  1.3 Briefly touch the 3 mm end of the PI wire with the pilot flame to burn off the insulation covering the PI wires. We will refer to this uninsulated portion as the 3 mm uninsulated end.
  1.4 Slightly open the oxygen valve at the top of the jewelry torch until the pilot flame has turned into a sharp blue flame. Opening the valve too fast or too much might cause a loud popping sound. If this occurs, close the oxygen valve, reignite the pilot flame, and repeat this step.
  1.5 Looking under the microscope, touch the end tip of the clamped 3 mm PI wire with the sharp blue flame to completely melt it into a ball. We will refer to it as the “big ball end” (Figure 8a).
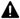
**CRITICAL STEP** Close the oxygen valve at the handle of the jewelry torch and place the jewelry torch in a secured location until further indication.
  1.6 Release the big ball end from the alligator clip and use a ruler to measure 7.6 cm of PI wire starting from the big ball end. Then, cut the wire at that location using the surgical scissors.
  1.7 Use a ruler to measure 6 mm of PI wire at the insulated end of the 7.6 cm PI wire (opposite from the big ball end) and clamp it with the micro-alligator clip.
  1.8 Burn off the insulation of the 6 mm clamped PI wire by briefly touching it with the pilot flame. We will refer to this uninsulated section as the 6 mm uninsulated end.
  1.9 Once again, slightly open the oxygen valve at the jewelry torch to turn the pilot flame into the sharp blue flame and melt 1 mm of PI wire at the tip of the clamped 6 mm PI wire. We will refer to the uninsulated 5 mm of PI wire and the 1mm melted ball collectively as the “small ball end” (Figure 8b). Completion of Steps 1–1.9 in this section will create a 7.5 cm wire that includes: a big ball end, a 6.7 cm insulated section (middle portion), and a small ball end.
  1.10 Repeat Steps 1–1.9 three more times to create a total of four longitudinal-cuff PI wires.
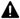
**CRITICAL STEP** Responsibly and safely disarm the jewelry torch set up after completion (make sure all valves are closed before dismantling).
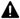
**CRITICAL STEP** Steps 1.11–1.17 must be performed under the microscope.
  1.11 Insert four gold pins into the female end of the dip socket.
  1.12 Use a paper clip to add a miniscule dab of water-soluble flux paste into the mounting holes of the four gold pins (Figure 8c).
  1.13 Set the temperature of the soldering iron to 700 °F and touch the top edge of each gold pin for approximately two seconds to melt the flux paste into the mounting holes. Repeat this step a couple more times to make sure the water-soluble flux has covered the entire inside of the gold pins.
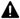
**CRITICAL STEP** Clean the soldering iron tip as needed using a wet sponge.
  1.14 One at a time, use the soldering iron to completely fill the mounting hole of each gold pin with lead-free solder (Figure 8d).
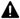
**CRITICAL STEP** No solder must drip from the mounting hole. If this occurs, replace the gold pin and repeat Steps 1.11–1.14.
  1.15 Use the tip of the #5 jeweler’s forceps to clean any excess solidified flux paste at the top of the gold pins.
  1.16 One at a time, touch the top edge each gold pin with the soldering iron to re-melt the solder inside the mounting hole. At the same time, insert the big ball end of the 7.5 cm PI wires (one for each gold pin). Then, remove the soldering iron from the gold pin and allow the solder to solidify with the big ball end inside the mounting hole.
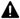
**CRITICAL STEP** Keep the wire straight and steady when inserting the big ball end of the PI wire into the mounting hole to prevent any damage to the insulation not being inserted.
  1.17 Apply a tiny drop of acrylic (liquid acrylic mixed with powder acrylic) at the top of each gold pin using a fine detail brush.
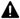
**CRITICAL STEP** No acrylic must drip off the top of the gold pins (Figure 8e). If this becomes the case, use a small paper towel to wipe off the acrylic around the gold pin.
2 **Cuff skeleton (5 min)**. Under the microscope, use a ruler to measure 4 mm of polyurethane tubing and cut it in a single straight scalpel cut.
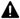
**CRITICAL STEP** The tube should have no rough edges at either end in order to prevent irritation in the animal after implantation.
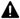
**CRITICAL STEP** This tube will be defined to be 4mm in length and a 360° surface (Figure 9a).
  2.1 Pass the closed tips of a #5 jeweler’s forceps through the inside of the 4mm polyurethane tube.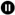 **PAUSE STEP** Dull forceps are recommended to prevent any possible stabbing injuries.
  2.2 Lay the forceps with the polyurethane tube flat against your working surface. Then, orientate the tube perpendicular to your shoulders.
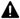
**CRITICAL STEP** The 4 mm polyurethane tube should be firmly stuck to the forceps. Make a single straight scalpel cut along the length of the tube at the face-up surface. We will refer to this as the anterior cut (Figure 9b).
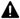
**CRITICAL STEP** Hold the #5 forceps tightly closed when making the cut to prevent a sudden opening of the forceps.
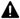
**CRITICAL STEP** Use the straight indentation between the tips of the #5 forceps to guide the scalpel across the surface of the tube.
  2.3 Use a fine-tip permanent marker to draw four points. The first mark will be located at one of the flat edges of the tube at a 0 mm length, and 45° to the right of the anterior cut. The remaining 3 marks should be drawn at the same flat edge 90° apart in a clockwise direction. We will refer to these as Marks B, D, F, and H respectively.
  2.4 Use the same marker to draw four more points at the opposite flat edge of the tube. These marks should be at reflected positions from Marks B, D, F, and H. We will refer to these marks as Marks A, C, E, and G (Figure 9c).
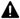
**CRITICAL STEP** Mark A must be at the opposite edge of the tube from Mark B, Mark C is opposite to Mark D, Mark E is opposite to Mark F, and Mark G is opposite to Mark H.
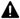
**CRITICAL STEP** Completing the cuff skeleton section will result in a 4mm tube containing an anterior cut and a total of eight marks (four at each flat end of the tube) (Figure 9c).
3 **Cuff assembly (20 min)**. Thread 30 cm of silk into the eye of the #5 sewing needle.
  3.1 Pierce the exterior surface of the tube with the silk-threaded needle 30° to the right of the anterior cut at a 1 mm length.
  3.2 Pass the entire needle through the puncture (and through the anterior cut) pulling the silk until approximately 3 cm is left on outside the tube. We will refer to this as the short silk end.
  3.3 Turn the needle around, pass it through the anterior cut, and pierce the inside surface of the tube 30° to the right of the anterior cut 2 mm above the puncture made in the previous step.
  3.4 Pass the entire needle through the puncture made in the previous step (towards the outside of the tube) until the silk between the two punctures has formed a “D” loop against the inside surface of the tube. The silk exiting the tube (with the needle at the tip) will be referred to as the long silk end.
  3.5 Make two ligature knots at the outside of the skeleton using the short silk end and the long silk end. See supplemental material for a detailed description of the ligature knotting technique.
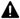
**CRITICAL STEP** All ligature knots must be firmly tied to prevent detachment during cuff assembly and after surgical implantation.
  3.6 Use a ruler to measure 6 cm of silk from the ligature knot towards the needle and make a cut with the surgical scissors at that location.
  3.7 Trim the 3 cm end to approximately 2 mm using the surgical scissors.
  3.8 Apply a miniscule drop of UV glue to the ligature knot with the eye of a clean #5 sewing needle.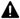 **CRITICAL STEP** The UV glue drop must be just big enough to cover the knot.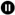 **PAUSE STEP** Put UV goggles on. UV goggles must be worn at all times as eye protection when using UV light.
  3.9 Cure the UV glue covering the ligature knot by shining UV light over it for 10 s.
  3.10 Use a tape strip to attach the 6cm silk strand to the flat working surface under the microscope.
  3.11 Repeat Steps 3.1 through 3.10 with the threaded needle at the left side of the anterior cut (instead of the right side as indicated by these steps). This will result in a total of two 6 cm silk strands connecting to their respective ligature knots (one on each side of the anterior cut) covered in solid UV glue taped to the working surface (Figure 10a).
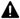
**CRITICAL STEP** The tube should lie perpendicular to your shoulders with the anterior cut facing up.
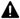
**CRITICAL STEP** The inside surface of the skeleton should be slightly visible through the anterior cut. If this is not the case, remove the tape covering one of the 6 cm silk strands, pull the silk, and reapply the tape.
  3.12 Use a clean #5 needle to pierce the exterior surface of the tube 0.5 mm directly above Mark A. Move ¾ of the needle into the tube (and through the anterior cut) leaving the last quarter on the outside of the initial puncture (Figure 10b).
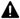
**CRITICAL STEP** Use your #5 forceps to press against the inner wall of the tube at that location to make the perforation easier.
  3.13 Pass the uninsulated end of a gold pin wire through the eye of the #5 needle piercing the tube (until half of the wire has gone through).
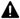
**CRITICAL STEP** Do not tie the gold pin wire to the eye of the needle or at any other point.
  3.14 Hold the gold pin end of the gold pin wire with the #5 forceps and pass the remaining quarter of the #5 needle through the puncture above Mark A (and through the anterior cut). Completing this step should move the uninsulated end of the gold pin wire towards the inside of the tube.
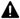
**CRITICAL STEP** The gold pin end of the gold pin wire should remain outside of the tube at all times.
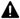
**CRITICAL STEP** Continue pulling until the gold pin wire has naturally slipped out of the eye of the needle.
  3.15 Pass the needle through the anterior cut and pierce the inside surface of the tube 0.5 mm below Mark B (at the top end of the tube). Move ¾ of the needle through the pierce leaving at least a quarter on the inside of the tube.
  3.16 Pass the uninsulated end of the gold pin wire through the eye of the needle (without looping it or knotting it) and pass the remaining quarter of the needle through the perforation thus pulling the gold pin wire towards the outside of the tube.
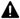
**CRITICAL STEP** A 3 mm uninsulated PI wire “D” loop should be formed against the inner wall of the tube between the punctures made between Mark A and B.
  3.17 Hold the small ball at the small ball end of the gold pin with the #5 forceps and the insulated portion of the PI wire next to the gold pin with your fingertips. Then, pull them in opposite directions to flatten the “D” loop at the inside of the tube.
  3.18 While keeping the “D” loop flat, retreat the gold pin wire through the punctures by pulling the gold pin end of the gold pin wire. Stop as soon as the last bit of the insulated section becomes aligned with the puncture above Mark A.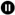 **PAUSE STEP** Put UV goggles on.
  3.19 Apply a small drop of UV glue to the puncture below Mark B using the needle in Step 3.8 to cover the small ball end of the gold pin wire exiting the tube. Then, cure the UV glue for ten seconds with the UV light.
  3.20 Repeat steps 3.11–3.19 with the remaining marks. Notice that Marks D, F, and H will be used instead of Mark B. Similarly, Marks C, E, and G will be used instead of Mark A (Figure 10c).
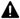
**CRITICAL STEP** The inside of the tube should now contain four 3 mm uninsulated leads 90° apart running across the length of the tube.
4 **Cuff Knotting (10–15 min).** Use the silk-threaded needle to pierce the exterior surface of the tube (and through the anterior cut) 15° to the right of the anterior cut at a 2 mm length. Continue pulling the needle until approximately 3 cm of silk is left on the outside. We will refer to the 3 cm silk piece as the short silk end.
  4.1 Turn with the needle 180°, pass it through the anterior cut, and pierce the inside surface medially, between the uninsulated A–B and C–D leads. Continue pulling the needle towards the outside of the tube until a “D” loop has formed at the inside of the tube.
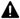
**CRITICAL STEP** The “D” loop should press the uninsulated lead running from Mark A to Mark B against the inside surface of the tube.
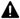
**CRITICAL STEP** The long piece of silk going towards the outside of the tube (with the needle at the end) will be referred to as the long silk end.
  4.2 Make a ligature knot at the outside of the tube using the short and long silk ends.
  4.3 Use the scissors to trim both ends of the ligature knot to approximately 3 mm.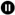 **PAUSE STEP** Use UV goggles as eye protection for the following steps.
  4.4 Apply a small drop of UV glue to the ligature knot with the eye of the needle and cure it by shining UV light over the glue for ten seconds. The UV glue drop should be small enough to only cover the knot, leaving the silk punctures to the right and left of the knot uncovered (preferably).
  4.5 Repeat Steps to 4–4.4 to create three more “D” loops (each pressing a lead against the inside surface of the tube) and three more ligature knots. However, instead of piercing at the location indicated in Step 4, use the last puncture of the previous knot to move the needle from the outside to the inside (and through the anterior cut) of the tube.
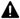
**CRITICAL STEP** It will only be necessary to make new punctures when moving the needle from the inside to the outside of the tube.
  4.6 One at a time, pull the insulated section of each gold pin wire above the gold pin to straighten the longitudinal uninsulated leads inside the tube, apply a small drop of UV glue at the B, D, F, and H punctures, and cure them under UV light for 15 s (Figure 10d).

## 4. Variable Multi-Contact Circumferential Cuff

**Figure 11:**
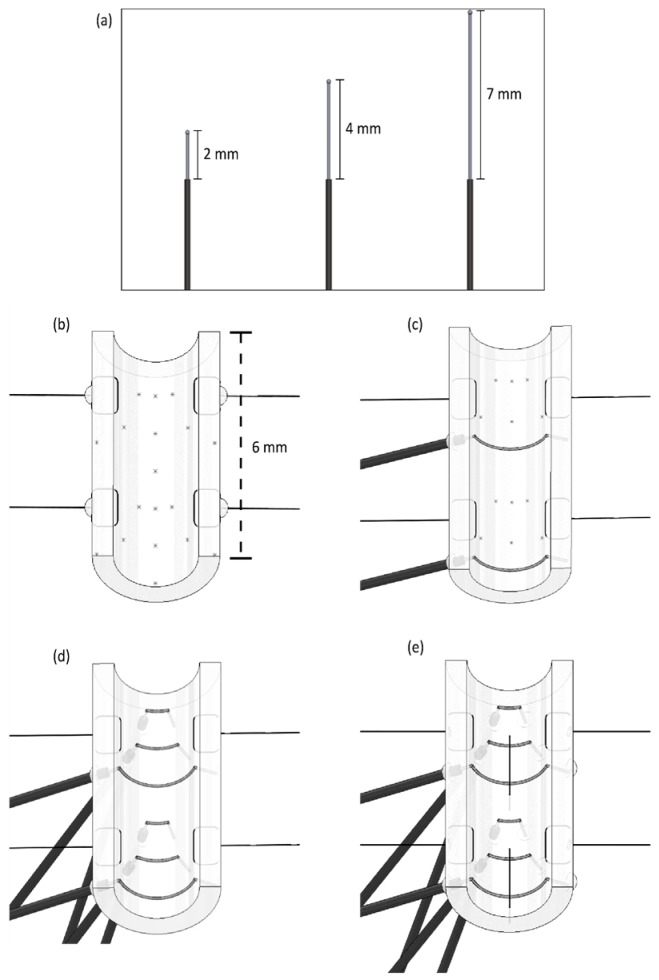
Variable Multi-contact Circumferential Cuff Micro-Construction Flowchart. (**a**) Illustration of PI wires with varying lengths of insulation removed. These wires will provide contact coverage of 60°, 120°, and 270° of the circumference of the cuff. (**b**) View of a 6mm segment of polyurethane tubing cut longitudinally and held open with silk strands. Marks for the 60°, 120°, and 270° coverage wires are depicted. (**c**) Cuff skeleton depicting the inserted 270° PI wires. (**d**) View showing insertion of the 120° and 60° PI wires. (**e**) Completed cuff, depicting exposed wires secured to the inside surface of the cuff with silk thread and all holes sealed with UV glue.

A detailed description of the Variable Multi-Contact Circumferential Cuff Electrode (Figure 11) construction process is presented below.

**Figure 12:**
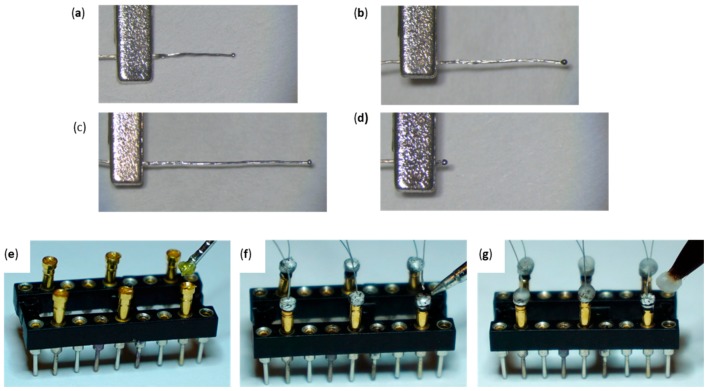
Variable Multi-contact Circumferential Cuff Gold Pin Wires Micro-construction; (**a**–**c**) Creation of the 60°, 120°, and 270° wires using the jewelry torch (**d**) big ball end of gold pin wires (same for all geometries). (**e**–**g**) Gold pin wire micro-construction using water-soluble flux paste, lead-free solder, and liquid/powder acrylic.

**Figure 13:**
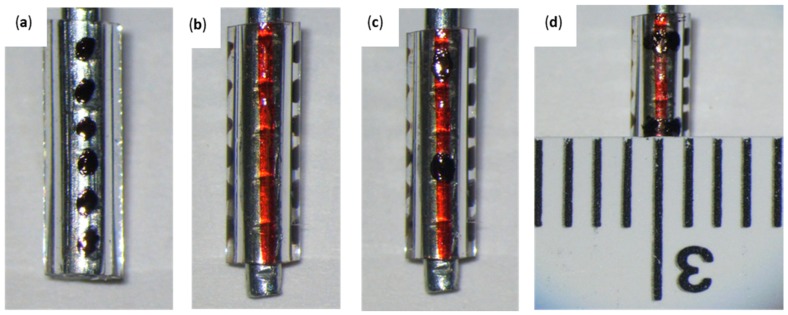
Variable Multi-contact Circumferential Cuff Skeleton Markings; (**a**) Posterior markings (1–6) (0.5 mm apart) (**b**) Frontal 0° line (red) across the 6 mm polyurethane tube. (**c**) Frontal view of the 6 mm polyurethane tube with the 5.5 mm mark (top) and the 2.5 mm mark (bottom). (**d**) Marking of two points 0.46 mm to the right and left of the upper and lower 270° lead marks using the space between two-millimeter marks and a centimeter mark of a ruler.

1 **60° Wire (5 min)**. Looking under the microscope, use a ruler to measure 3 mm of platinum-iridium (PI) wire and hold it in the micro-alligator clip. Do not detach or cut the wire from the wire spool.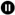 **PAUSE STEP** Flammable materials required ahead. Previous experience using a jewelry torch is recommended. Do not ignite the pilot flame pointing at yourself or other individuals.
  1.1 Slightly open the propane valve at the torch handle and ignite the pilot flame by placing the flame of a lighter near the tip of the torch.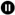 **PAUSE STEP** Performing Step 1.2 may dangerously heat the micro-alligator clip and the PI wire. Always allow approximately 1 minute for cool down before touching the clip and the wire after these steps.
  1.2 Briefly touch the 3 mm end of the PI wire with the pilot flame to burn off the insulation covering the PI wires. We will refer to this uninsulated portion as the 1 mm uninsulated end.
  1.3 Slightly open the oxygen valve at the top of the jewelry torch until the pilot flame has turned into a sharp blue flame. Opening the valve too fast or too much might cause a loud popping sound. If this occurs, close the oxygen valve, reignite the pilot flame, and repeat this step.
  1.4 Melt 1 mm of PI wire at the tip of the clamped 3 mm PI wire by touching it with the sharp blue flame. We will refer to the uninsulated 2 mm of PI wire and the 1mm melted ball collectively as the “small ball end” (Figure 12a).
  1.5 Release the small ball end of the 60° PI wire from the alligator clip and use a ruler to measure 7.5 cm of PI wire from the tip of the 2 mm uninsulated end to the spool of PI wire. Then, cut the wire at that location using the surgical scissors.
  1.6 USe a ruler to measure 3 mm of PI wire at the insulated end of the 7.5 cm PI wire (opposite from the small ball end). Then, hold it in the micro-alligator clip.
  1.7 Completely melt the 3 mm locked PI wire by touching it with the sharp blue flame. We will refer to this end as the big ball end (Figure 12d). Completion of steps 1–1.7 in this section will create a 7.2 cm wire with a 2 mm uninsulated end (small ball end), a 7 cm insulated section (middle portion), and a big ball end.
  1.8 Repeat Steps 1–1.7 to create a second 7.2 cm PI wire.
2 **120° Wire (5 min).** Looking under the microscope, use a ruler to measure 5 mm of platinum-iridium (PI) wire and hold it in the micro-alligator clip. Do not detach or cut the wire from the wire spool.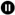 **PAUSE STEP** Performing Step 2.1 may dangerously heat the micro-alligator clip and the PI wire. Always allow approximately 1 min for cool down before touching the clip and the wire after these steps.
  2.1 Briefly touch the 5mm end of the PI wire with the pilot flame to burn off the insulation covering the PI wires. We will refer to this uninsulated portion as the 5 mm uninsulated end.
  2.2 Slightly open the oxygen valve at the top of the jewelry torch until the pilot flame has turned into a sharp blue flame. Opening the valve too fast or too much might cause a loud popping sound. If this occurs, close the oxygen valve, reignite the pilot flame, and repeat this step.
  2.3 Melt 1 mm of PI wire at the tip of the clamped 5 mm PI wire by touching it with the sharp blue flame. We will refer to the uninsulated 4mm of PI wire and the 1 mm melted ball collectively as the “small ball end” (Figure 12b).
  2.4 Release the small ball end of the 120° PI wire from the alligator clip and use a ruler to measure 7.7 cm of PI wire from the tip of the small ball end to the spool of PI wire. Then, cut the wire at that location using the surgical scissors.
  2.5 Use a ruler to measure 3 mm of PI wire at the insulated end of the 7.7 cm PI wire (opposite from the small ball end). Then, hold it in the micro-alligator clip.
  2.6 Completely melt the 3 mm locked PI wire by touching it with the sharp blue flame. We will refer to this end as the big ball end (Figure 12d). Completion of Steps 2–2.6 in this section will create a 7.4 cm wire with a 4 mm uninsulated end (small ball end), a 7 cm insulated section (middle portion), and a big ball end.
  2.7 Repeat Steps 2–2.6 to create a second 7.4 cm PI wire.
3 **270° Wire (5 min)**. Looking under the microscope, use a ruler to measure 8 mm of platinum-iridium (PI) wire and hold it in the micro-alligator clip. Do not detach or cut the wire from the wire spool.
  3.1 **PAUSE STEP** Performing Step 3.1 may dangerously heat the micro-alligator clip and the PI wire. Always allow approximately 1 minute for cool down before touching the clip and the wire after these steps.
    Briefly touch the 8 mm end of the PI wire with the pilot flame to burn off the insulation covering the PI wires. We will refer to this uninsulated portion as the 8 mm uninsulated end.
  3.2 Slightly open the oxygen valve at the top of the jewelry torch until the pilot flame has turned into a sharp blue flame. Opening the valve too fast or too much might cause a loud popping sound. If this occurs, close the oxygen valve, reignite the pilot flame, and repeat this step.
  3.3 Melt 1 mm of PI wire at the tip of the clamped 8 mm PI wire by touching it with the sharp blue flame. We will refer to the uninsulated 7 mm of PI wire and the 1 mm melted ball collectively as the “small ball end” of the 270° PI wire (Figure 12c).
  3.4 Release the PI wire small ball end from the alligator clip and use a ruler to measure 8 cm of PI wire from the small ball end to the spool of PI wire. Then, cut the wire at that location using the surgical scissors.
  3.5 Use a ruler to measure 3 mm of PI wire at the insulated end of the 8 cm PI wire (opposite from the small ball end). Then, hold it in the micro-alligator clip.
  3.6 Completely melt the 3 mm locked PI wire by touching it with the sharp blue flame. We will refer to this end as the big ball end (Figure 12d). Completion of Steps 3–3.6 in this section will create a 7.7 cm wire with a 7 mm uninsulated end (small ball end), a 7 cm insulated section (middle portion), and a big ball end.
  3.7 Repeat Steps 3–3.6 to create a second 7.7 cm PI wire.
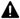
**CRITICAL STEP** Steps 4–4.7 must be performed under the microscope.
4 **Gold Pin Wires (60°, 120°, and 270°) (10 min).** Firmly secure the male end of the gold pin dip socket to the permeable surface. Do this by pushing it against the surface aid until the gold pin dip socket has pierced the surface.
  4.1 Insert six gold pins into the female end of the dip socket.
  4.2 Use a paper clip to add a miniscule dab of water-soluble flux paste into the mounting holes of the six gold pins (Figure 12e).
  4.3 Set the temperature of the soldering iron to 700 °F and touch the top edge of each gold pin for approximately two seconds to melt the flux paste into the mounting holes. Repeat this step a couple more times to make sure the water-soluble flux has covered the entire inside of the gold pins.
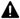
**CRITICAL STEP** Clean the soldering iron tip as needed using a wet sponge.
  4.4 One at a time, use the soldering iron to completely fill the mounting hole of each gold pin with lead-free solder.
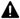
**CRITICAL STEP** No solder must drip from the mounting hole. If this occurs, replace the gold pin and repeat Steps 4.1–4.4.
  4.5 Use the tip of the #5 jeweler’s forceps to clean any excess solidified flux paste at the top of the gold pins.
  4.6 One at a time, touch the top edge of each gold pin with the soldering iron to re-melt the solder inside the mounting holes. At the same time, insert the big ball end of the PI wire (one for each gold pin). Then, remove the soldering iron from the gold pin and allow the solder to solidify with the big ball end inside the mounting hole (Figure 12f).
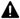
**CRITICAL STEP** Keep the wire straight and steady when inserting the big ball end of the PI wire into the mounting hole to prevent any damage to the insulation not being inserted.
  4.7 Apply a tiny drop of acrylic (liquid acrylic mixed with powder acrylic) at the top of each gold pin using a fine detail brush.
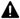
**CRITICAL STEP** No acrylic must drip off the top of the gold pins (Figure 12g). If this becomes the case, use a small paper towel to wipe off the acrylic around the gold pin.
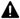
**CRITICAL STEP** All procedures in this section must be performed under a microscope.
5 **Cuff skeleton (10–15 min)**. Use a ruler to measure 6mm of polyurethane tubing and cut it using a scalpel. We will treat this tube as a cylinder of 6mm in length.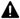 **CRITICAL STEP** Make a single straight cut when performing this step. The tube should have no rough edges at either end in order to prevent irritation in the animal.
  5.1 Insert the tip of one 1 ¾” paper clip into the 6mm polyurethane tube and lay them horizontally over your working surface. Orient the polyurethane tube perpendicular to your shoulders. We will refer to the current face-up surface of the 6mm tube as the posterior side.
  5.2 USe a fine-tip permanent marker to draw six points along the length of the 6 mm tube. The first point should be drawn 0.5 mm above the bottom edge of the tube and will be referred to as Point 1. The remaining points should be drawn 1 mm apart in a straight line towards the top edge of the tube and referred to as Points 2–6 (Figure 13a).
  5.3 Move the posterior side of the tube face-down by rotating the 6 mm polyurethane tube 180° at the tip of the paper clip. Then, use a fine-tip marker and a ruler to draw a straight line along the entire length of the tube directly across from Points 1–6. We will refer to this as the 0° line (Figure 13b).
  5.4 USe the permanent marker and the ruler to draw a point at a 2.5 mm length from the bottom end of the tube on the 0° line. This point should be exactly across the tube from Point 3 located at the posterior surface (Figure 13c).
  5.5 Draw a point at a 5.5 mm length from the bottom of the tube on the 0° line using the fine-tip permanent marker and the ruler. This point should be exactly across the tube from Point 6 (Figure 13c).
  5.6 Place the ruler directly over the point drawn at a 2.5 mm length in a perpendicular position and align any one of the centimeter marks with the tube’s 0° line. The ruler should cover half of the point when placed over it. A ruler with distinct centimeter lines compared to the millimeter lines is required for visual aid purposes.

USe the permanent fine-tip marker to indicate two points 0.46 mm to the right and left of the point drawn at a 2.5 mm length. Use the space between the centimeter line and the adjacent millimeter marks of the ruler to approximate the correct location. A little less than half way between the centimeter mark and the adjacent millimeter marks of the ruler may be used as a visual approximation. Use a caliper to verify your placement once the points have been drawn. We will refer to the two marks as the lower right and left 270° lead marks.

5.7 Repeat Steps 5.6 and 5.7 at the point drawn at a 5.5 mm length. We will refer to the two marks as the upper right and left 270° lead marks (Figure 13d).
5.8 Rotate the tube 180° on the paper clip bringing the posterior surface face-up.
5.9 Place the ruler over Point 1 in a perpendicular position and align one of the centimeter marks with the point. The body of the ruler should cover only half of the point.
5.10 Draw two points 0.31 mm to the right and left of Point 1. Use the space between the centimeter line and the adjacent millimeter marks of the ruler to visually approximate the correct location and use a caliper to verify the placement. We will refer to these points as the lower 60° lead marks.
5.11 Repeat Steps 5.10 and 5.11 for Point 4. We will refer to the new marks as the upper 60° lead marks.
5.12 Place the ruler over Point 2 and align it with a centimeter mark. The ruler should cover only half the point when placed over.
5.13 Use the fine-tip marker to indicate two points 0.61 mm to the right and left of the Point 2. Use the space between the centimeter line and the adjacent millimeter marks of the ruler to visually approximate the correct location. We will refer to the two marks as the lower right and left 120° lead marks.
5.14 Repeat Steps 5.13 and 5.14 over Point 5, located at a 4.5 mm length from the bottom edge of the tube. We will refer to the two marks sideways from Point 5 as the upper right and left 120° lead marks.
5.15 Turn the tube 180° on the paper clip to move the anterior side of the tube face-up. Then, make a single-straight scalpel cut along the entire 0° line of the tube. We will refer to this as the anterior cut.

**Figure 14:**
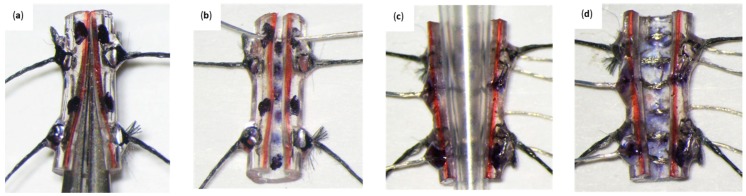
Variable Multi-contact Circumferential Cuff Micro-construction; (**a**) Attachment of silk strands 90° to the left and right of the 6mm polyurethane tube. Knots are located at a 5 mm (top knots) and 1 mm (bottom knots) length. (**b**) Insertion of the 270° gold pin wire for the longitudinal cuff at the upper 270° lead marks using a needle. (**c**) 5 µL pipette tip pressing the top and bottom 270°, 120°, and 60° uninsulated gold pin wire portions against the inside wall of the 6mm polyurethane tube. (**d**) Frontal upside-down view of the Variable Multi-contact Circumferential Cuff. Image location of the 60° electrodes (first and fourth in a top-down order), 120° electrodes (second and fifth in a top-down order), and 270° electrodes (third and sixth in a top-down order).

6 **Cuff assembly (20–30 min)**. Thread 30 cm of silk into the eye of the #5 sewing needle.
  6.1 Pierce the exterior surface of the tube with the silk-threaded needle 90° to the right of the anterior cut and at a 5 mm tube length from the bottom edge of the tube.
  6.2 Pass the entire needle through the pierce and pull the silk until approximately 3 cm are left on outside the tube. We will refer to this as the short silk end.
  6.3 Turn the needle around, pass it through the anterior cut, and pierce the inside surface of the tube 90° to the right of the anterior cut 1 mm below the previous puncture (Step 6.1). Pass the entire needle through the puncture and pull the needle until the silk between the two pierces has formed a “D” loop against the inside surface of the tube. The silk exiting the tube (with the needle at the tip) will be referred to as the long silk end.
  6.4 Make two ligature knots at the outside of the skeleton using the short silk end and the long silk end. See supplemental material for a detailed description of the ligature knotting technique.
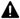
**CRITICAL STEP** All ligature knots must be firmly tied to prevent detachment during cuff assembly and after surgical implantation.
  6.5 Use a ruler to measure 6 cm of silk from the ligature knot towards the needle and make a cut using the surgical scissors at that location.
  6.6 Trim the 3 cm end to approximately 2 mm using the surgical scissors.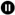 **PAUSE STEP** Put UV goggles on. UV goggles must be worn at all times as eye protection when using UV light.
  6.7 Apply a miniscule drop of UV glue to the ligature knot with the eye of a clean #5 sewing needle.
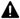
**CRITICAL STEP** The UV glue drop must be just big enough to cover the knot.
  6.8 Cure the UV glue covering the ligature knot by shining UV light over it for 10 s.
  6.9 Use a tape strip to attach the 6 cm silk strand to the flat working surface under the microscope perpendicular to the tube.
  6.10 Repeat Steps 6.2–6.9 with the threaded needle 90° to the right of the anterior cut. The D loop should start at a 2 mm length and exit the tube at a 1 mm length.
  6.11 Repeat Steps 6.2–6.10 at the left side of the tube. Use the same measurements to create mirror ligature knots on the left side of the tube.
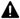
**CRITICAL STEP** Completion of these steps will result in a total of four ligature knots with two on each side of the tube. (Figure 14a).
  6.12 Pass the uninsulated end of one of the 270° gold pin wires through the eye of a #5 sewing needle.
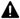
**CRITICAL STEP** Do not loop or knot the wire to the needle at any point.
  6.13 Hold the gold pin end of the gold pin wire with the #5 forceps and pierce the exterior surface of the tube at the right upper 270° lead mark with the needle.
  6.14 Pass the needle through the puncture until reaching the inner wall of the tube across the left 270° upper lead mark and pierce the inner wall of the tube at that location.
  6.15 Pass the entire needle towards the outside of the tube until the uninsulated end of the gold pin wire has slipped out of the eye (Figure 14b).
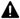
**CRITICAL STEP** Hold the gold pin end of the gold pin wire at the outside of the tube while performing step 6.15.
  6.16 Insert the 10 µL pipette tip through the anterior cut to press the gold pin wire against the inside wall of the tube.
  6.17 Slowly retract the gold pin wire by pulling the gold pin end at the right side of the tube until only uninsulated wire lies against the inside of the tube.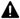 **CRITICAL STEP** If needed, trim the small ball end of the gold pin wire to approximately 1–2 mm.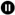 **PAUSE STEP** Put UV goggles on.
  6.18 Apply a small drop of UV glue to the puncture at the left 270° upper mark using the eye of a clean needle and cure it for ten seconds under UV light.
  6.19 Repeat Steps 6.12–6.18 using the second 270° gold pin wire at the right and left lower 270° marks.
  6.20 Release the upper two silk strands from the tape and flip the 6 mm tube to move the posterior side facing up. There is no need to reattach the silk strands to the working surface.
  6.21 Insert the uninsulated end of one of the 120° gold pin wires through the eye of the needle.
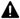
**CRITICAL STEP** Do not loop or knot the wire to the needle at any point.
  6.22 Use the needle to pierce the exterior surface of the tube at the upper right 120° mark towards the anterior cut.
  6.23 Pass the needle completely through the puncture leaving the gold pin end of the gold pin wire at the outside of the tube.
  6.24 Make a U-turn with the needle and use it to pierce the interior wall of the tube directly across the upper left 120° mark. Once the needle is completely through the puncture, release the gold pin wire from its eye.
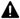
**CRITICAL STEP** The gold pin wire should form a D loop at the inside of the tube between the right and left upper 120° marks.
  6.25 Insert the pipette tip through the anterior cut to press the gold pin wires at the inside of the tube (Figure 14c).
  6.26 Retract the 120° gold pin wire by pulling the gold pin end at the right upper 120° mark until only the uninsulated portion remains on the inside of the tube.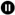 **PAUSE STEP** Put UV goggles on.
  6.27 Apply a small drop of UV glue to the puncture at the left upper 120° mark using the eye of a clean needle and cure it for ten seconds under UV light. Then, remove the pipette tip at the inside of the tube.
  6.28 Repeat Steps 6.21–6.27 with the second 120° gold pin wire at the right and left lower 120° marks.
  6.29 Repeat Steps 6.21–6.28 using the two 60° gold pin wires at the upper and lower right and left 60° marks (Figure 14d).
7 **Cuff Knotting (10 min)**. Use the silk-threaded needle to pierce the exterior surface of the tube (and through the anterior cut) 0.5 mm below the upper 270° mark at the posterior side of the tube. Continue pulling the needle until approximately 3 cm of silk are left on the outside. We will refer to the 3cm silk piece as the short silk end.
  7.1 Turn the needle 180°, pass it through the anterior cut, and pierce the inside surface of the tube approximately 0.5 mm below the upper 60° electrode. Continue pulling the needle towards the outside of the tube until a “D” loop has formed at the inside of the tube covering the 270° lead and the 120° lead.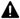 **CRITICAL STEP** The long piece of silk going towards the outside of the tube (with the needle at the end) will be referred to as the long silk end.
  7.2 Make a ligature knot at the outside of the tube using the short and long silk ends.
  7.3 Use the scissors to trim both ends of the ligature knot to approximately 3 mm.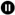 **PAUSE STEP** Wear UV goggles for the following steps.
  7.4 Apply a small drop of UV glue to the ligature knot with the eye of a clean needle and cure it by shining UV light over the glue for ten seconds. The UV glue drop should be only big enough to cover the knot.
  7.5 Repeat Steps 7–7.4 0.5 mm below the lower 270° mark and 0.5 mm below the lower 60° electrode at the inside of the cuff.
  7.6 Place the pipette tip inside the tube to press the uninsulated segment of the PI wires against the inner surface of the tube.
  7.7 Apply a small drop of UV glue to the right upper and lower 270°, 120°, and 60° punctures with exiting PI wire and cure them under UV light for 10 s. This is now a completed Variable Multi-contact Circumferential Cuff.
  7.8 **VNS Cuff Impedance Testing (15–20 min)**
    1 Fill a small container with saline.
    2 Connect the test probe pins male plugs to the LCR meter.
    3 Hold the gold pins of the cuff in the test probe pins of the LCR meter.
    4 Submerge the tube of the cuff completely in the saline.
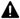
**CRITICAL STEP** Do not submerge the probes with the pins.
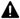
**CRITICAL STEP** Visually ensure that saline fills the inside of the cuff.
    5 Turn on the LCR meter and set the frequency to 1 kHz, then observe the impedance value displayed on the screen. Functional VNS cuffs should have impedance of less than 2 kΩ.

If the cuff contains more than two gold pins, switch the gold pins on the probe pins of the LCR meter and record their impedance.

## 5. Expected Results

**Figure 15:**
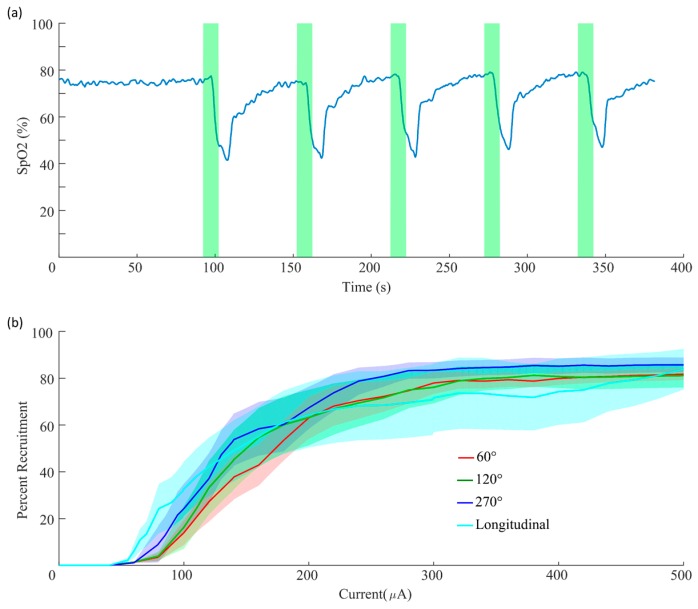
Oxygen Saturation Reductions and Sciatic Nerve Dose-Response Curves; (**a**) Representative Oxygen Saturation Data During Repeated VNS; Blue line indicates oxygen saturation levels in the animal’s blood over time. Green vertical bars represent vagus nerve stimulation at multiple time points. (**b**) Average Sciatic Fiber Recruitment Functions for All Cuff Styles; shaded area represents error of the mean.

We used two methods to confirm the efficacy of nerve stimulation using cuffs constructed using the above described protocols. First, to evaluate activation of the vagus nerve, we measured rapid stimulation-dependent depression of blood oxygen saturation, an effect ascribed to the Hering–Breuer reflex [26]. Stimulation of vagal A-fibers, which include the pulmonary stretch receptors, generates a feedback suppression of inhalation and causes blood oxygen saturation to transiently fall (Figure 15a). Therefore, measurement of oxygen saturation provides a simple means to assess vagal A-fiber recruitment. As expected, 10 s trains of stimulation (0.8 mA, 100 µs biphasic pulses, 30 Hz) delivered using the standard nerve cuff model generate robust, reliable drops blood oxygen saturation, signaling activation of the vagus nerve (Figure 15a). 

We next tested activation of the sciatic nerve using the longitudinal and multichannel cuffs. Stimulation of the A-fibers in the sciatic nerve triggers contraction of the gastrocnemius muscle, thus assessment of the degree of force generated by the gastrocnemius provides a simple means to evaluate A-fiber recruitment. As expected, stimulation delivered across a range of stimulation intensities (0.5 s train, 0–0.5 mA, 100 µs biphasic pulses, 30 Hz) using either the longitudinal and multichannel circumferential cuffs both produced effective recruitment of the nerve at low stimulation intensities (Figure 15b). Together, these data indicate that the cuff construction methods above provide reliable vagal and sciatic nerve stimulation. 

## Figures and Tables

**Table 1:** Required equipment for cuff micro-construction.

Required Equipment	Provider	Catalog Number
BD General Use and PrecisionGlide Hypodermic Needles (22G 1)	Fisher Scientific	14-826B
BD General Use and PrecisionGlide Hypodermic Needles (27G 1¼)	Fisher Scientific	14-826-48
8in. Bastard-Cut Mill File	Home Depot	21832
Student Scalpel Handle—#3	Fine Science Tools	91003-12
#11 Sterile Surgical (Scalpel) Blade	Fisher Scientific	08-915-18
BESSEY 4 in. × 2 in. Bar Clamp	Home Depot	LM2.004
Micro-Gator CL Steel	Mueller Electric	548-34
Measuring Ruler	Fisher Scientific	S40641
Bernzomatic Oxygen 1.4OZ Oxygen Gas Cylinder	Home Depot	304179
Bernzomatic 14.1 oz Propane Cylinder	Walmart	1139232
Gentec Small Torch Basic Kit with Easy Turn Knobs, For Oxy/Propane	Kingsley North Inc.	6-1447
Student Fine Scissors	Fine Science Tools	91460-11
DIP/SIP Socket	Mouser Electronics	110-93-316-41-001000
Weller WES51 Analog Soldering Station	Amazon	3439-01-176-0339
Student Dumont #5 Forceps	Fine Science Tools	91150-20
#5 Sewing Needle	Dritz	74217
Scotch Greener Masking Tape for Basic Painting, 0.94 in × 60.1 yd (24 mm × 55 m)	Walmart	2025-24C
Thermo Scientific™ ART™ Barrier Hinged Rack Pipette Tips	Fisher Scientific	02-682-254
3.5X-90X Trinocular LED Boom Stand Stereo Microscope with 144-LED and Auto Focus Camera	AmScope	SM-3TZ-144S-AF1
Refillable Butane Lighter—2/Pack	Webstaurant Store	22311006
Smart Nickel Finished Non-Skid Paper Clips, Silver, 1.25”, 100-Pack	Walmart	563296067
Helping Third Hand Magnifier W/Magnifying Glass Tool—MZ101	Adafruit	291
Size 2/0—Alan Johnson Signature Fine Detail Long Striping Brush	TCP Global	MAC AJ-2/0
Surgical Weck-Cell Spears	Medline	8680
Smith Preset propane/mapp gas regulator (Red)	Contenti	114-273-01
Smith Preset oxygen regulator (Green)	Contenti	114-273-02
Medline Isopropyl Rubbing Alcohol 70%	Five Star Supply	MDS098003Z
RX 0.9% Sodium Chloride for Injection 1000 mL IV Bag	Med-Vet	RX0.9NACL-K
BK Precision® 879B LCR meter	Mouser Electronics	615-879B
Beaker Low Form Glass Graduated 100 mL Bomex	Amazon	B00122DP6G
Mini Hook Test Leads, 24 In. L, Black/Red	Grainger	3782-24-02
Loctite 7700 Hand Held LED Light Source, 100 to 240 VAC, 47/63 Hz, 3 W	Amazon	1427231-30769
ACCO® Economy Metal Paper Clips, No. 1, Silver, 100 Per Pack, Box Of 10	Office Depot	619785
Digital Caliper	KC Tool	41101

**Table 2:** Critical components for cuff micro-construction.

Critical Components	Provider	Catalog Number
Platinum–Iridium Multistrand Wire (1 ft)	Sigmund Cohn	10IR9/49T
Press-Fit Pins Gold Connectors “Gold Pins”	Mouser Electronics	1001-0-15-15-30-27-04-0
AIM Solder SN100C-GLOW-2.5%-020-1LB Glow Core No-Clean Wire Solder, 1 lb Spool	Amazon	477-690
H-20®5 Water-Soluble Paste Flux 1.7 oz	Home Depot	301302
Kiss Acrylic Fill Kit	Walmart	AK105
Micro-Renathane Tubing Per Ft. 0.037” × 0.023” Continuous Length (36 ft)	Braintree Scientific	MRE037
6/0 Braided Silk Suture Thread (0.08 mm Diameter)	Fine Science Tools	18020-60
Loctite AA 3106 (Loctite 3106)	Henkel Adhesives	23697

## Supplementary Materials

The following are available online at https://www.mdpi.com/2409-9279/2/1/19/s1. See Supplementary Ligature Knot Video.
